# Potassium-Mediated Variations in the Photosynthetic Induction Characteristics of *Phaseolus vulgaris* L.

**DOI:** 10.3390/plants14111623

**Published:** 2025-05-26

**Authors:** Qi Luo, Wei Jin, Lili Li, Kedong Xu, Yunmin Wei

**Affiliations:** 1 Key Laboratory of Plant Genetics and Molecular Breeding, Zhoukou Normal University, Zhoukou 466001, China; luoqi@zknu.edu.cn (Q.L.); lilili@zknu.edu.cn (L.L.); 2 Henan Plant Gene and Molecular Breeding Engineering Research Center, Zhoukou Normal University, Zhoukou 466001, China; jinw877@126.com; 3 College of Life Science and Agriculture, Zhoukou Normal University, Zhoukou 466001, China

**Keywords:** photosynthetic induction, potassium, photosynthetic limitation, *Phaseolus vulgaris* L., maximum carboxylation rate

## Abstract

Plants are commonly exposed to fluctuating illumination under natural light conditions, causing dynamic photosynthesis and further affecting plant growth and productivity. In this context, although the vital role of potassium (K) in steady-state photosynthesis has been well-established, knowledge of the dynamic changes in photosynthesis mediated by K remains scarce. Here, the gas-exchange and chlorophyll fluorescence parameters under steady-state and dynamic photosynthetic responses were quantified in *Phaseolus vulgaris* L. seedlings grown under K-deficient (−K, 0.02 mM K) and normal K (+K, 2 mM K) conditions. After a transition from low to high light, the time course–induction curves of the net photosynthetic rate (*A*), stomatal conductance (*g*_s_), mesophyll conductance (*g_m_*), and maximum carboxylation rate (*V*_cmax_) showed an obvious decline in the −K treatment. In comparison with the +K treatment, however, there were no statistical differences in the initial *A* and *V*_cmax_ values in *P. vulgaris* supplied with deficient K, suggesting that the K-deficiency-induced decreases in *A* and *V*_cmax_ were light-dependent. Interestingly, the time to reach 90% of the maximum *A*, *g*_s_, and *g*_m_ significantly decreased in the −K treatment in comparison with the +K treatment by 27.2%, 45.6%, and 52.9%, respectively, whereas the time to reach 90% of the maximum *V*_cmax_ was correspondingly delayed by almost two-fold. The photosynthetic limitation during the induction revealed that the biochemical limitation was the dominating factor that constrained *A* under the −K conditions, while, under the +K conditions, the main limiting factor changed from biochemical limitation to stomatal limitation over time. Moreover, *g*_m_ imposed the smallest limitation on *A* during induction in both K treatments. These results indicate that a decreased K supply decreases the photosynthetic performance under fluctuating light in *P. vulgaris* and that improving the induction responses of biochemical components (i.e., *V*_cmax_) has the potential to enhance the growth and productivity of crops grown in K-poor soil.

## 1. Introduction

Potassium (K), alongside nitrogen and phosphorus, is the most important macronutrient for plant growth. In particular, it participates in many essential physiological processes, e.g., photosynthesis, osmotic regulation, and stomata regulation [1,2]. Consequently, K deficiency is widely recognized as a major limiting factor on photosynthetic carbon assimilation and ultimately impairs plant productivity [3]. In recent years, steady-state photosynthesis under optimal environmental conditions has become a major focus for elucidating how K deficiency limits photosynthetic carbon assimilation, as K-deficiency-induced photosynthesis stress in a leaf is a dynamically aggravated process [4]. Moreover, increasing evidence indicates that the reduction in the net photosynthetic rate (*A*) under K-limited conditions cannot be ascribed solely to stomatal limitations and is instead due to the integrated contribution of stomatal, mesophyll, and biochemical (carboxylation-related) factors [5,6,7].

Potassium plays an essential role in regulating guard cell turgor [8], and a deficiency or an inadequate supply of K results in a decrease in stomatal conductance (*g*_s_), thereby reducing CO_2_ diffusion into the leaf chloroplast [9]. However, even if g_s_ is partially maintained under these conditions, the photosynthetic capacity remains suboptimal [10,11], suggesting that additional resistances are involved. Mesophyll conductance (*g*_m_) from the sub-stomatal cavities to the carboxylation sites inside the chloroplast stroma has emerged as a major constraint of *A* under K stress. According to Lu et al. [12], K starvation causes disadvantages in leaf structure alterations among different plant species, such as a reduced internal air space, a decreased exposed surface area of chloroplasts in intercellular airspaces, and an increased distance from the chloroplast to the cell wall, which largely impede internal CO_2_ diffusion and finally reduce g_m_. In addition, K is essential for the activation of enzymes in the Calvin–Benson cycle, particularly the rubisco enzyme [13]. K-starved plants often exhibit rubisco activity reduction, delayed RuBP regeneration, and disrupted stromal ion homeostasis, which ultimately contribute to declined carboxylation efficiency [14,15]. Overall, elucidating these relative contributions of limiting factors is crucial for optimizing K management strategies to sustain high crop productivity.

However, while a lot of supporting evidence has been found in the literature to explain the effect of K deficiency on the steady-state photosynthesis of plants, such as wheat [16], rice [17], oilseed rape [14], and maize [18], plants in natural or semi-natural ecosystems are rarely exposed to controlled, steady-state conditions. Instead, environmental factors, especially irradiance, change rapidly (over seconds to minutes throughout the day) due to variable solar position, cloud movement, or self-shading under canopies [19]. Plants therefore frequently encounter rapid fluctuations in light intensity, especially in seasons when the weather is changeable. Under these conditions, the capacity of photosynthetic systems to adjust, which is termed “dynamic photosynthesis”, is critical for enhancing daily carbon gain by plants [20]. A previous study showed that faster stomatal opening and the activation of electron transport persevere as the most effective targets for increasing photosynthetic responses by plants under dynamic light conditions [21], which may be largely determined by K ions. However, the role of K in dynamic photosynthesis responses has not yet been fully explored. Therefore, it is essential to understand how K affects the rate and flexibility of photosynthetic responses to changeable environmental conditions.

Similar to steady-state photosynthesis, dynamic photosynthesis is also controlled by *g*_s_, *g*_m_, and the biochemical capacity (i.e., the maximum carboxylation rate, *V*_cmax_), which are generally downregulated under K-deficient conditions [22,23]. However, the integrated mechanisms that cause the variation in dynamic photosynthesis by K deficiency are complex, and the relative contributions of stomatal, mesophyll, and biochemical limitations during light transitions have not been clearly defined. It is speculated that the increase in the relative ratios of *g*_s_, *g*_m_, or *V*_cmax_ under K deficiency reflect an excess capacity relative to photosynthetic system demands, potentially facilitating faster induction. On the contrary, these unchanged or decreased ratios may imply slower photosynthetic responses. This raises critical questions as to whether K-deficient plants exhibit delayed photosynthetic induction and, more importantly, which components (stomatal, mesophyll, or biochemical factors) exert the predominant limiting factor under such conditions.

To address these questions, we used common bean (*Phaseolus vulgaris* L.) as an experimental material to investigate the impact of potassium deficiency on both steady-state and dynamic photosynthesis. Specifically, the objectives of this study were to (1) evaluate the effects of K deficiency on steady-state photosynthetic performance; (2) characterize the dynamic variations in *A*, *g*_s_, *g*_m_, and *V*_cmax_ during photosynthetic induction; and (3) quantify the dominant constraint on photosynthesis during induction using a time-integrated limitation analysis.

## 2. Results

### 2.1. Photosynthetic Parameter Variations During Photosynthetic Induction

**Figure 1:**
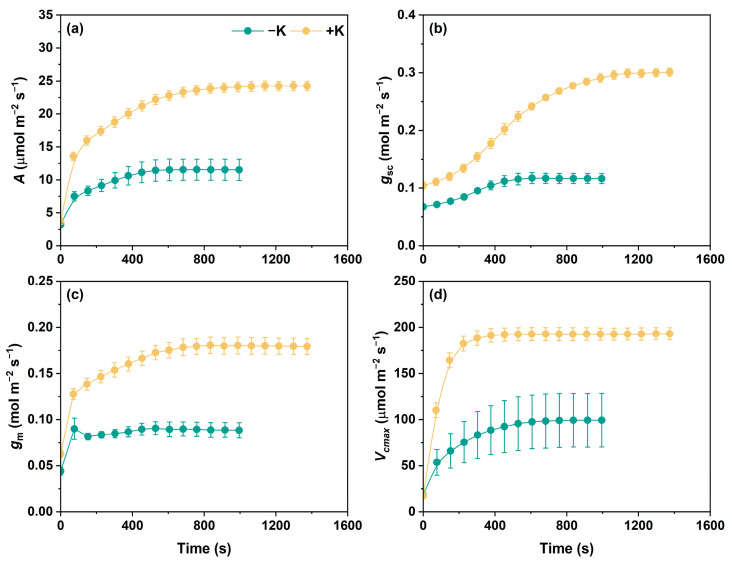
Dynamic photosynthetic traits of *Phaseolus vulgaris* L. during photosynthetic induction at 1000 μmol m^−2^ s^−1^ photosynthetic photon flux density (PPFD) as affected by K nutrition. Leaves were initially acclimated to a steady state under low light (100 μmol m^−2^ s^−1^ PPFD), followed by exposure to high light (1000 μmol m^−2^ s^−1^ PPFD). *A*, net photosynthetic rate (**a**); *g*_sc_, stomatal conductance to CO_2_ (**b**); *g*_m_, mesophyll conductance to CO_2_ (**c**); *V*_cmax_, the maximum carboxylation rate (**d**).

As demonstrated by the curves in Figure 1, the leaf net photosynthetic rate (*A*), stomatal conductance (*g*_s_), mesophyll conductance (*g*_m_), and, particularly, the maximum carboxylation rate (*V*_cmax_) rapidly responded when *P. vulgaris* was exposed to sudden light irradiation. Specifically, the induction curves of *A* under both potassium-deficient (−K) and normal potassium (+K) conditions showed typical logarithmic increases, and *A* was markedly higher under +K than under −K throughout the induction period after the low-to-high light transition. Interestingly, *P. vulgaris* L. under the −K conditions reached steady state faster than that under the +K conditions, within almost 15 min, with this increasing to more than 20 min under the +K conditions. The induction of *g*_sc_, *g*_m_, and *V*_cmax_ showed almost the same responses as *A*. However, it is worth noting that the improvement in *g*_sc_ and g_m_ under −K was quite limited. In comparison with the initial *g*_sc_, the increase in *g*_sc_ at the end of the induction period was 42% under −K and 211% under +K. As for photosynthetic system II (PSII), the actual phytochemical efficiency of PSII (ΦPSII) was substantially higher in the +K treatment than in the −K treatment (Figure S1).

### 2.2. Steady Changes in Photosynthetic Traits During Photosynthetic Induction

**Figure 2:**
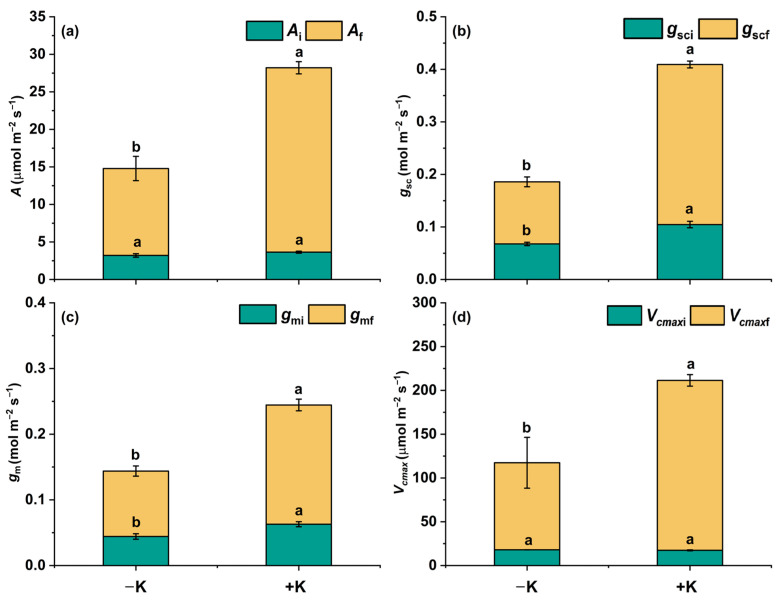
Steady-state photosynthetic parameters of *Phaseolus vulgaris* L. after light photosynthetic induction as affected by K nutrition. *A*_i_ (**a**), *g*_sci_ (**b**), *g*_mi_ (**c**), and *V*_cmaxi_ (**d**) are the steady-state *A*, *g*_sc_, *g*_m_, and *V*_cmax_ of the last 1 min of the low-light induction period (100 μmol m^−2^ s^−1^ PPFD). *A*_f_ (**a**), *g*_scf_ (**b**), *g*_mf_ (**c**), and *V*_cmaxf_ (**d**) are the steady-state *A*, *g*_sc_, *g*_m_, and *V*_cmax_ of the last 1 min of the high-light induction period (1000 μmol m^−2^ s^−1^ PPFD). Different letters indicate significant differences between the treatments (*p* < 0.05). Data are presented as means ± SE (n = 5).

There were no statistical differences between the −K and +K treatments in terms of either *A*_i_ or *V*_cmaxi_ after low-light induction (100 PPFD); nevertheless, both *g*_si_ and *g*_mi_ were significantly higher under the +K conditions than under the −K conditions, increasing by approx. 54.4% and 42.3% (Figure 2). For *P. vulgaris* L. exposed to 1000 PPFD light induction, the steady-state photosynthetic parameters *A*_f_, *g*_scf_, *g*_mf_, and *V*_cmaxf_ were significantly higher under the +K treatment than under the −K treatment, among which K had the greatest effect on *g*_scf_. Compared with the −K treatment, *g*_scf_ increased by about 158% under the +K treatment (Figure 2).

### 2.3. Dynamic Changes in Photosynthetic Traits During Photosynthetic Induction

**Figure 3:**
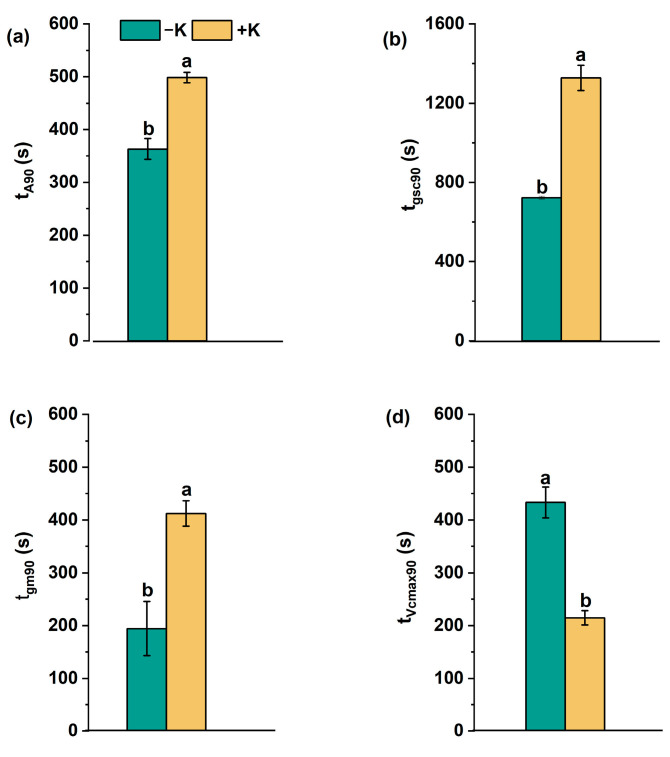
Dynamic photosynthetic parameters of *Phaseolus vulgaris* L. during photosynthetic induction as affected by K nutrition. t_A90_ (**a**), t_gsc90_ (**b**), t_gm90_ (**c**), and t_vcmax90_ (**d**) are the times to reach 90% photosynthetic induction, full stomatal opening, mesophyll opening, and biochemical activation. Different letters indicate significant differences between the treatments (*p* < 0.05). Data are presented as means ± SE (n = 5).

In general, during the light induction period (Figure 1), most photosynthetic parameters under the −K treatment were found to approach 90% of full induction more rapidly than under the +K treatment (Figure 3). Specifically, for *A*, the time to 90% maximum (t_A90_) was approx. 363 s compared to 498 s, and, for *g*_sc_ and *g*_m_, the time to 90% maximum under +K was 83.7% (t_gsc90_) and 112.1% (t_gm90_) longer than that under −K. On the contrary, *V*_cmax_ required a significantly longer time to reach the 90% maximum (t_vcmax90_) under the −K treatment, being almost 2-fold longer than under the +K treatment.

### 2.4. Temporal Responses of Photosynthetic Limitations During Photosynthetic Induction

**Figure 4:**
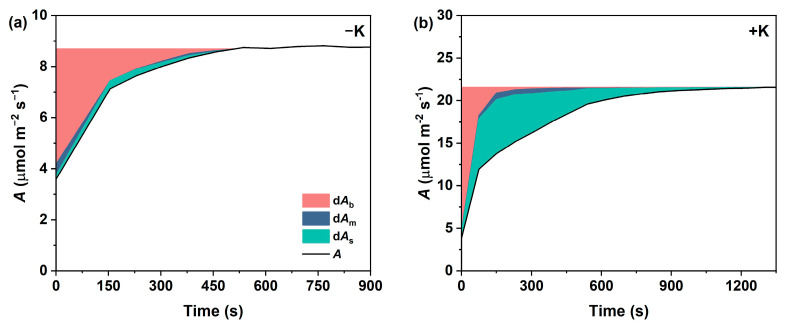
Dynamic variations in the biochemical limitation (d*A*_b_), mesophyll conductance limitation (d*A*_m_), and stomatal limitation (d*A*_s_) of *Phaseolus vulgaris* L. during photosynthetic induction under K-deficient condition (**a**) and normal K condition (**b**).

To further examine the contributions of *g*_sc_, *g*_m_, and *V*_cmax_ to *A* during photosynthetic induction, the deviation in A (d*A*) at a given time point was quantitatively partitioned into components corresponding to stomatal (d*A*_s_), mesophyll (d*A*_m_), and biochemical (d*A*_b_) limitations. As shown in Figure 4, in the first 300 s of induction under the −K treatment, the d*A*_b_ values accounted for the largest proportion of the total limitation, in comparison with d*A*_s_ and d*A*_m_. However, in *P. vulgaris* L. treated with +K, the relative biochemical limitation was initially almost 100%, then it decreased rapidly after illumination, and it gradually decreased to 0% after 300 s. On the contrary, during the induction period, d*A*_s_ increased gradually and became the main limiting factor of d*A* after 2 min. Moreover, the d*A*_m_ values were the lowest throughout the entire induction period in both the −K and +K treatments, despite subtle differences being observed between the treatments; i.e., in the −K treatment, mesophyll limitation occurred in the initial stages of the induction, while, in the +K treatment, it occurred in the mid-to-late stages.

**Figure 5:**
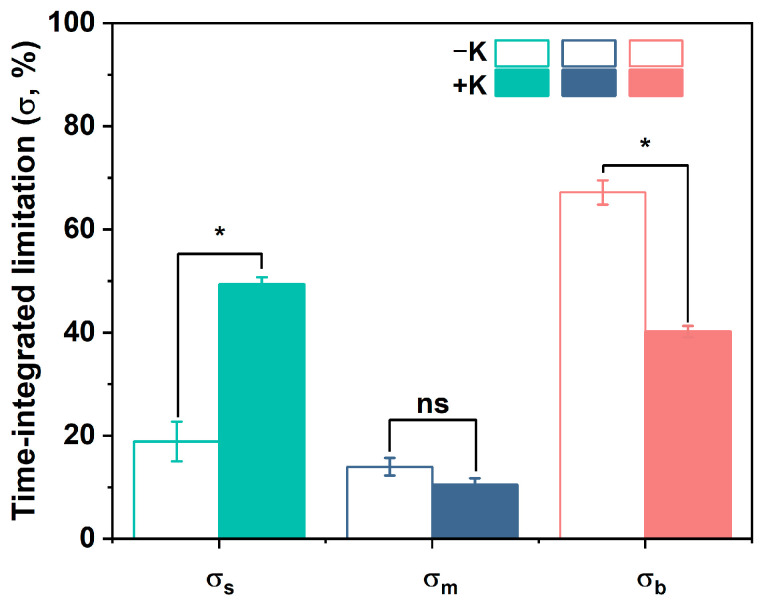
Impact of K nutrition on the relative time-integrated limitations involving stomatal limitation (σ_s_), mesophyll conductance limitation (σ_m_), and biochemical limitation (σ_b_) in response to the photosynthetic induction of *Phaseolus vulgaris* L. Data are presented as means ± SE (n = 5). Asterisks indicate significant differences between the treatments (*, *p* < 0.05; ns, no significant difference).

When integrating the relative limitations of photosynthesis over the entire induction period, it was observed that the K supply significantly affected the main photosynthetic limiting factor of *P. vulgaris* L., despite there being no statistical differences in σ_m_ between the −K and +K treatments (Figure 5). Under the −K conditions, the biochemical limitation (σ_b_) was the strongest limiting factor of photosynthesis, accounting for 67.2% of the total photosynthetic limitation. Meanwhile, under the +K conditions, the stomatal limitation (σ_s_) was found to account for 49.4% of the total limitation, followed by the biochemical limitation (40.2%) and mesophyll limitation (10.5%).

## 3. Discussion

Previous studies have demonstrated the general presence of weakened photosynthetic carbon assimilation under K-deficient or inadequate conditions [12,24,25]. In the present study, the steady-state *A* (*A*_f_ as an indicator) of *P. vulgaris L*. in the −K treatment declined by 52.9% in comparison with A in normal K supply. In addition to the steady-state *A* under high light, the photosynthetic responses to changes in illumination significantly affect the carbon gain of plants [26,27]; the average *A* value under the −K conditions during the photosynthetic induction process was 10 μmol m^−2^ s^−1^, statistically lower than that under the +K conditions (average of 20.8 μmol m^−2^ s^−1^), with an average decline of 51.0%. It is worth noting that the time required for *A* to reach a steady state in −K was significantly shorter than that in +K, despite there being a decrease in the *A* value under the −K conditions. Similar trends were observed in the response curves for *g*_sc_, *g*_m_, and *V*_cmax_ during the induction.

According to Kaiser [20], the time to reach 90% *A* of the full induction (t_A90_) is defined as the photosynthetic response rate, which is mainly influenced by nutrition, plant species, and stress factors. For instance, tomatoes supplied with high nitrogen levels showed a much faster photosynthetic induction response than those supplied with moderate or low nitrogen levels [28]; however, in *Panax notoginseng*, a higher nitrogen content in leaves caused a slower photosynthetic induction rate [29]. Zhang et al. [30] found that tomato exposed to salt stress demonstrated a significantly reduced response rate, resulting in an increased t_A90_. These findings indicate that plants under nutrient or other stress conditions have a prolonged photosynthetic induction time, which results in an increased *t*_A90_. However, in our current results, the *t*_A90_ values decreased by approx. 27.2% in the K-deficient treatment compared to in the normal K treatment.

It is generally accepted that the actual t_A90_ is a comprehensive result influenced by t_gs90_, t_gm90_, and t_Vcmax90_. As shown in Figure 3, the t_gs90_ and t_gm90_ of plants supplied with deficient K were significantly shorter than those of plants supplied with normal K nutrition. However, not all plant responses occurred within the same timescale; *A* and *g*_m_ responded and reached 90% of steady state within 5–8 min, whereas changes in *g*_s_ took more than 10 min (Figure 3), in accordance with previous studies [31,32,33]. Therefore, it was supposed that the lower t_gsc90_ under the −K conditions may be mainly responsible for the rapid t_A90_. As *g*_sci_ was not higher in the −K treatment than in the +K treatment as expected, it is logical to believe that the K deficiency induced stomatal closure, thereby decreasing t_gsc90_ during the irradiation. Stomatal movement occurs with channel-mediated K^+^ uptake over time [32] due to the vital role of potassium in the osmotic mechanism of stomata aperture modulation. During the photosynthetic induction, the light-dependent influx and efflux of potassium of guard cells could have been affected by the K deficiency and, thus, regulated stomatal closure [34,35]. Similar results were obtained in studies on tea [36], *Eucalyptus grandis* [37], rice [38], and olive [39]. Further studies are needed to analyze the dynamic responses of stomatal movement mediated by K supplement during photosynthetic induction. Moreover, K is essential for increasing enzyme activity in higher plants [40,41]. According to a study on *Brassica napus*, a decrease in K decreased the rubisco content and activity by 37% and 58.4%, respectively, causing a decreased carboxylation rate [42]. In the present study, although there were no statistical differences regarding the initial *V*_cmax_ between treatments, the final state of *V*_cmax_ was significantly reduced in the −K treatment compared to in the +K treatment and was accompanied by a longer t_Vcmax90_ (Figure 2 and Figure 3).

A sufficient understanding of photosynthetic limiting factors during light fluctuation is of relevance to solving the substantial decline in daily carbon accumulation in plants [21,43]. A recent study by Liu et al. [22] demonstrated the important role of *g*_m_ in limiting *A* during photosynthetic induction. However, another study on Arabidopsis and tobacco found opposite results, showing a transition from a stomatal-dominating limitation to a biochemical limitation during induction [28]. In the present study, in *P. vulgaris* L. plants supplied with deficient K, photosynthetic limitations due to *g*_s_, *g*_m_, and biochemical factors changed slightly upon the transition from low to high light, in which condition *A* was mainly constrained by biochemical factors, while, under the normal K conditions, the predominating photosynthetic limiting factors gradually changed from biochemical limitation to stomatal limitation during the induction process. Meanwhile, *g*_m_ consistently imposed the smallest limitation on A between the K treatments. As it turns out, the limiting factors of photosynthetic induction changed frequently, induced by external environmental conditions, plant species, or genotypes [22,44,45].

As evident in Figure 5, under both the −K and +K conditions, *g*_m_ was identified as being the least limiting of the three photosynthetic components on a time-integrated scale, while the biochemical capacity (i.e., *V*_cmax_) was the most significant limiting factor of photosynthetic induction for the plants supplied with deficient K. In contrast to our findings, Lu et al. [12] found that the *g*_m_ limitation contributed to more than one-half of the *A* decline under K-deficient conditions. In this regard, this may reflect the adaptive strategies of plants to −K stress at different time scales, as several processes underlying photosynthesis activate or deactivate cooperatively [46]. However, although a reduced *g*_sc_ was observed in the K-deficient conditions, it takes a certain amount of time for stomatal opening to occur in photosynthetic induction, whereas the activation of the rubisco enzyme usually occurs faster (several seconds to minutes) [47,48]. However, as K^+^ plays a critical role in the biosynthesis and activation of rubisco, the deficient K supply resulted in an increased t_Vcmax90_ and, finally, constrained *A*, especially during the short period of photosynthetic induction.

## 4. Materials and Methods

### 4.1. Plant Material

A hydroponic culture experiment was conducted in a plant culture room with an illuminated light source maintained at approx. 800 μmol m^−2^ s^−1^ photosynthetic photon flux density (PPFD). The photoperiod was set to 16 h at 25 °C per day and 18 °C per night, and the relative humidity was 50–60%. *P. vulgaris* seeds were germinated in moist filter papers at 4 °C for 12 h, which were then fixed to a transit box for germination. After 7 d, uniform seedlings were transferred to 10 L plastic containers and supplied with one-quarter-strength nutrient solution (for the composition, see below). Five days later, the seedlings were supplied with one-half-strength nutrient solution. After another 5 d, the seedlings were supplied with full-strength nutrition with K-deficient (−K, 0.02 mM K_2_SO_4_) or normal K treatment (+K, 2 mM K_2_SO_4_). The composition of the full-strength nutrition solution was as follows: 5 mM N with mixed (NH_4_)_2_SO_4_ and Ca(NO_3_)_2_, 1mM NaH_2_PO_4_·2H_2_O, 2 mM K_2_SO_4_, 2.5 mM CaCl_2_, 1 mM MgSO_4_, 0.05 mM Fe-EDTA, 10 μM H_3_BO_3_, 10 μM MnCl_2_·4H_2_O, 0.3 μM CuSO_4_·5H_2_O, 0.8 μM ZnSO_4_·7H_2_O, and 0.01 μM Na_2_MoO_4_·2H_2_O. The pH of the nutrient solution was adjusted to 7.0 ± 0.1 every day and was completely changed every 3 d.

### 4.2. Measurement of Gas Exchange and Fluorescence

Two weeks after the treatments started, fully expanded leaves of each plant were selected for the measurement of gas-exchange parameters from 9:00 to 11:30, using a portable gas-exchange system (Li-6400XT, Li-Cor, Lincoln, NE, USA). The PPFD was set to 1000 μmol m^−2^ s^−1^ (red light–blue light, 90%:10%), the vapor pressure deficit (VPD) was between 1.4 and 1.6 Kpa, the air flow rate was 500 μmol s^−1^, the leaf temperature was 25 ± 0.3 °C, and the CO_2_ concentration was maintained at 400 ± 10 μmol mol^−1^. Once steady state was achieved, the steady-state fluorescence yield (*F*_s_) and maximum fluorescence (*F*_m_′) were recorded with a light-saturating pulse (0.8 s) of 8000 μmol m^−2^ s^−1^; in the meantime, the net photosynthetic rate (*A*), CO_2_ concentration in intercellular spaces (*C*_i_), and leaf stomatal conductance (*g*_s_) were recorded. As *g*_s_ is the stomatal conductance to water vapor, it is expressed in terms of stomatal conductance to CO_2_ (*g*_sc_) in subsequent calculations, which was calculated as *g*_sc_ = *g*_s_/1.6.

### 4.3. Estimation of Mesophyll Conductance (g_m_)

The actual phytochemical efficiency of PSII (ΦPSII) was then calculated as follows:

ΦPSII = (*F*_m_′ − *F*_s_)/*F*_m_′ (1)

The liner electron transfer rate (J) was given as

J = ΦPSII × PPFD × α × β(2)

where α is the leaf absorption and β is the proportion of quanta absorbed by PSII, assumed to be 0.85 and 0.5, respectively. Taken together, the variable J method was used to calculate *g*_m_ [49], given as follows:

(3) $$g_{m}=\frac{A}{C_{i}-\frac{\Gamma^{*}(J+8(A+R_{d}))}{J-4(A+R_{d})}}$$

(4) $$C_{c}=C_{i}-\frac{A}{g_{m}}$$

where Γ* is the CO_2_ compensation point in the absence of mitochondrial respiration and *R*_d_ is the mitochondrial respiration rate in the light. Γ* was assumed to be 40.0 μmol m^−2^ s^−1^, and *R*_d_ was assumed to be 1.0 μmol m^−2^ s^−1^. Using the above equation, *g*_m_ could be calculated at each time point.

### 4.4. Calculations of the Maximum Carboxylation Rate of Rubisco (V_cmax_)

According to Mott et al. [50], plants can maintain saturated RuBP under both low- and high-light irradiance conditions; therefore, rubisco activation was modulated to limit *A* for the transition from low to high irradiation. It was then assumed that the Rubisco limitation would adequately constrain *A* throughout photosynthetic induction. *V*_cmax_ was determined as follows:

(5) $$V_{\mathrm{cmax}}=\frac{\left(A+R_{d}){(C}_{i}+K_{m}\right)}{\left(C_{i}-\Gamma^{*}\right)}$$

where *R*_d_ is the rate of respiration in the light, and *K*_m_ is the effective CO_2_ Michaelis–Menten constant for rubisco. Here, *K*_m_ was set to 509.5 at 25 °C. Using the above equation, *V*_cmax_ could be calculated at each time point.

### 4.5. Determination of Photosynthetic Induction Parameters

For induction, leaves were initially acclimated to a steady state under low light (100 μmol m^−2^ s^−1^ PPFD) for 30 min, followed by exposure to high light (1000 μmol m^−2^ s^−1^ PPFD) until the photosynthetic parameters approached a steady state. Gas-exchange measurements were recorded every second for the first minute and every five seconds thereafter. Plants were randomly selected and measured from 9:00 to 11:30. Measurements were repeated 4 times on individual replicates for each treatment over a span of 2 days to reduce the time effects. For each treatment, 5 biological replicates were used, and all calculations were performed on single replicates.

Photosynthetic induction was calculated according to the following equations, where the subscripts “i” and “f” refer to the steady-state assimilation rate (*A*) values in the last minute of low light and high light, respectively:

(6) $$\mathrm{IS}=\frac{A-A_{i}}{A_{f}-A_{i}}$$

The induction of *g*_sc_, *g*_m_, and *V*_cmax_ over the same duration was also calculated by replacing *A* with *g*_sc_, *g*_m_, and *V*_cmax_, respectively.

Moreover, t_A90_, t_gsc90_, t_gm90_, and t_Vcmax90_ were defined as the time required to obtain 90% of the difference between the initial and maximum values of photosynthetic induction, stomatal opening, mesophyll conductance, and the maximum values of V_cmax_, respectively.

### 4.6. Limitation Analysis

The relative changes in light-saturated assimilation can be expressed in terms of the relative changes in stomatal, mesophyll conductance, and biochemical capacity, Hence, the *A* variation can be modeled using

(7) $$\frac{dA}{A}={dA}_{s}+{dA}_{m}+{dA}_{b}$$

where d*A*_stom_, d*A*_mes_, and d*A*b_iochem_ are the stomatal, mesophyll conductance, and biochemical limitations on *A*, which can be determined using

(8) $${dA}_{s}=\frac{\partial A}{{\partial g}_{\mathrm{sc}}}dg_{\mathrm{sc}}$$

(9) $${dA}_{m}=\frac{\partial A}{{\partial g}_{m}}dg_{m}$$

(10) $${dA}_{b}=\frac{\partial A}{{\partial V}_{\mathrm{cmax}}}dV_{\mathrm{cmax}}$$

where d*g*_sc_, d*g*_m_, and d*V*_cmax_ are the variations between the final state and steady-state *g*_sc_, *g*_m_, and *V*_cmax_, respectively.

Given that *A* = *g*_sc_ (*C*_a_ − *C*_i_), solving the partial derivatives combined with Equation (1) gives

(11) $$\frac{\partial A}{{\partial g}_{\mathrm{sc}}}=\frac{\frac{A}{g_{\mathrm{sc}}^{2}}\left((V_{\mathrm{cmax}}-R_{d}\right)-A)}{{(V}_{\mathrm{cmax}}-R_{d})(\frac{1}{g_{\mathrm{sc}}})+(C_{a}+K_{m})-2(\frac{1}{g_{\mathrm{sc}}})A}$$

(12) $$\frac{\partial A}{{\partial g}_{m}}=\frac{\frac{A}{g_{m}^{2}}\left((V_{\mathrm{cmax}}-R_{d}\right)-A)}{{(V}_{\mathrm{cmax}}-R_{d})(\frac{1}{g_{\mathrm{sc}}})+(C_{a}+K_{m})-2(\frac{1}{g_{\mathrm{sc}}})A}$$

(13) $$\frac{\partial A}{{\partial V}_{\mathrm{cmax}}}=\frac{\left(C_{a}-\Gamma^{*}\right)-A(\frac{1}{g_{\mathrm{sc}}})}{{(V}_{\mathrm{cmax}}-R_{d})(\frac{1}{g_{\mathrm{sc}}})+(C_{a}+K_{m})-2(\frac{1}{g_{\mathrm{sc}}})A}$$

### 4.7. Statistical Analysis

All statistical analyses were carried out using SPSS 25.1. A one-way ANOVA was performed to evaluate the significant differences in the parameters between groups. Graphical depiction was conducted with Origin Pro 2020. Data are presented as mean ± standard error (SE).

## 5. Conclusions

In the current study, we examined the effects of K supplementation on photosynthetic induction after transfer from low to high light in *P. vulgaris* L., and the induction curves of *A*, *g*_sc_, *g*_m_, and *V*_cmax_ showed significant differences during the induction process. Regarding the time course of photosynthesis induction, t_A90_, t_gm90_, and t_gsc90_ were shorter under the −K conditions than under the +K conditions, while the induction time of *V*_cmax_ was significantly delayed, which further caused a lag in changes in t_A90_ under the K-deficient conditions. During the transition from low to high light, increasing the induction responses of *V*_cmax_ may have the potential to improve *A* in *P. vulgaris* plants, especially when they are grown under low-K conditions, whereas the photosynthetic induction of *A* was rarely limited by *g*_m_ under both the −K and +K treatments. Taken together, the present study demonstrates the important role of biochemical capacities in limiting *A* during photosynthetic induction. Therefore, improving biochemical-related parameters, such as rubisco enzyme activity, is likely an effective strategy for improving dynamic photosynthetic performance in *P. vulgaris* under K-deficient conditions.

## Data Availability

Data are contained within the article.

## Supplementary Materials

The following supporting information can be downloaded at: https://www.mdpi.com/article/10.3390/plants14111623/s1, Figure S1: Actual phytochemical efficiency of PSII photosystem (ΦPSII) of *Phaseolus vulgaris* L. during photosynthetic induction at 1000 μmol m^−2^ s^−1^ photosynthetic photon flux density (PPFD) as affected by K nutrition.

## Abbreviations

*A*	net photosynthetic rate
*g*_sc_	stomatal conductance to CO_2_
*g*_m_	mesophyll conductance to CO_2_
*V*_cmax_	maximum carboxylation rate
ΦPSII	actual phytochemical efficiency of PSII
*A*_i_	steady-state A during the last 1 min of low-light induction period
*g*_si_	steady-state g_sc_ during the last 1 min of low-light induction period
*g*_mi_	steady-state g_m_ during the last 1 min of low-light induction period
*V*_cmaxi_	steady-state V_cmax_ during the last 1 min of low-light induction period
*A*_f_	steady-state A during the last 1 min of high-light induction period
*g*_sf_	steady-state g_sc_ during the last 1 min of high-light induction period
*g*_mf_	steady-state g_m_ during the last 1 min of high-light induction period
*V*_cmaxf_	steady-state V_cmax_ during the last 1 min of high-light induction period
t_A90_	time for A to reach 90% of photosynthetic induction
t_gs90_	time for g_sc_ to reach 90% of photosynthetic induction
t_gm90_	time for g_m_ to reach 90% of photosynthetic induction
t_Vcmax90_	time for V_cmax_ to reach 90% of photosynthetic induction
d*A*_b_	transient biochemical limitation during photosynthetic induction
d*A*_m_	transient mesophyll conductance limitation during photosynthetic induction
d*A*_s_	transient stomatal conductance limitation during photosynthetic induction
σ_b_	time-integrated biochemical limitation during photosynthetic induction
σ_m_	time-integrated mesophyll conductance limitation during photosynthetic induction
σ_s_	time-integrated transient stomatal conductance limitation during photosynthetic induction
