# Supplementary material for: Potassium-Mediated Variations in the Photosynthetic Induction Characteristics of Phaseolus vulgaris L
Source: Plants (Basel). 2025 May 26;14(11):1623. doi: 10.3390/plants14111623 (PMC12156992; doi:10.3390/plants14111623)
Supplement: Supplementary file 1 [file plants-14-01623-s001.zip › plants-3627827-supplementary.pdf]

Supplementary data

Title: Potassium mediated variations of photosynthetic induction characteristics of *Phaseolus vulgaris* L.

Authors: Qi Luo[1,2], Wei Jin[2,3], Lili Li[1,3], Kedong Xu[1,2*], and Yunmin Wei[1,2,*]

Figure S1 Actual phytochemical efficiency of PSII photosystem (ΦPSII) of *Phaseolus vulgaris L.* during photosynthetic induction at 1000 μmol m^{-2} s^{-1} photosynthetic photon flux density (PPFD) as affected by K nutrition.

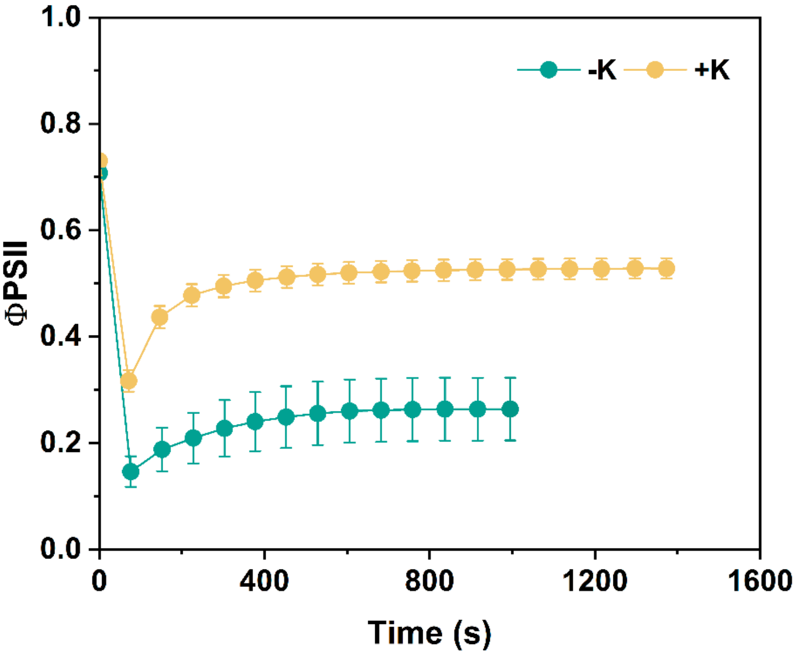

Figure S1. Actual phytochemical efficiency of PSII photosystem (ΦPSII) of *Phaseolus vulgaris* L. during photosynthetic induction at 1000 µmol m^{-2} s^{-1} photosynthetic photon flux density (PPFD) as affected by K nutrition. Leaves were initially acclimated to a steady state under low light (100 µmol m^{-2} s^{-1} PPFD), followed by an exposure to high light (1000 µmol m^{-2} s^{-1} PPFD).
